# Understanding the influencing factors and mechanism of social compensation for Chinese older adults using social media in the context of smart home: a qualitative analysis

**DOI:** 10.3389/fpubh.2023.1174920

**Published:** 2023-10-12

**Authors:** Ke Ma, Meng Gao, Francesco Ermanno Guida, Renke He

**Affiliations:** ^1^School of Design, Hunan University, Changsha, China; ^2^Design Department, Politecnico di Milano, Milan, Italy

**Keywords:** compensation theory, social media, social compensation, smart screen technology for smart homes, Chinese older adults, grounded theory, qualitative analysis, users’ acceptance behavior

## Abstract

As a new generation of necessary terminals for future homes, smart homes have become one of the essential mediums for smart aging at home. This paper aims to explore how older adults who age at home can overcome the digital divide of the new medium and achieve social participation in the home context to realize active aging. Based on the theory of social compensation, we select the smart-home smart screen, a representative new medium product in China, and carry out open coding, spindle coding, selective coding, and theoretical construction of the original interview data through the grounded theory research method. The results show that the main factors affecting the social compensation of older adults to smart home social media include user interface quality, interaction quality, content quality, and service quality, and these four factors are used as external variables to compensate older adults socially, thereby stimulating the emotional experience and perception changes at the cognitive level of older adults and then affecting the adoption and acceptance of smart home social media by older adults. This study refines the factors influencing the older adults’ use of smart home social media from the perspective of social compensation. It explains the mechanism of acceptable behavior of older adults, bridging the gap in previous literature on the influencing factors and behavioral mechanisms of older adults of smart home social media. This paper provides a theoretical basis and guidance for the subsequent academic research and software development practice of social media under new technological devices to further help older adults in China achieve active and healthy aging.

## 1. Introduction

China is in the midst of an aging and digitalized society. On the one hand, China’s aging problem is severe, and the seventh national census of China, released in May 2021, shows that 18.7% of the total population, or 264 million people, are aged 60 and over, up 5.44% from the sixth census (1). Among them, the number of older adults living alone and empty nesters increased to 118 million, and it is expected that the number of empty nesters will exceed 200 million by 2030 (2). At the same time, the lower-aged older adult population aged 60–69 accounts for 55.83% of the total number of older adults, or about 10.44% of the total population (3). This is a significant increase in the number of older adults in China. The current situation in China is that the aging population continues to rise, with a large proportion of older adults living alone, empty nesters, and a high proportion of young older adults. To solve the problem of aging the first step is to solve the problem of old-age care. Since the pension model adopted in China is a “90-7-3” home-based care pattern (4), 90% of older adults aged 60 or above live at home. The remaining 10% choose daycare home-based services, and only a few attend institutions. Thus, more than 90% of older adults in China live at home. The main issue confronting a large number of older adults living alone and empty nesters is how to meet their social participation needs. Social participation is a fundamental factor that affects whether older adults can live independently and actively (5). Older people themselves are also willing to spend time maintaining contact with family and close friends (6). However, due to the decline in physical functions, mobility difficulties, retirement from the workforce, shrinking social circles, the impact of the post-epidemic era, as well as the need to cope with the sadness caused by widowhood and the loss of loved ones and friends, the above personal and social factors can lead to a lack of social interaction between the older adults and the rest of society, resulting in social isolation. They may feel lonely and emotionally unsupported, which can be psychologically damaging. Loneliness is associated with and synergistic with depression, reducing older adults’ well-being (7). Loneliness also increases the risk of death in older adults (8). Studies have shown that social interaction and participation in social activities can not only help alleviate social isolation and loneliness in older adults (9), but also help reduce the risk of Alzheimer’s disease (10). Therefore, how encouraging the social participation of older adults living alone at home has become an important practical issue.

**Figure 1:**
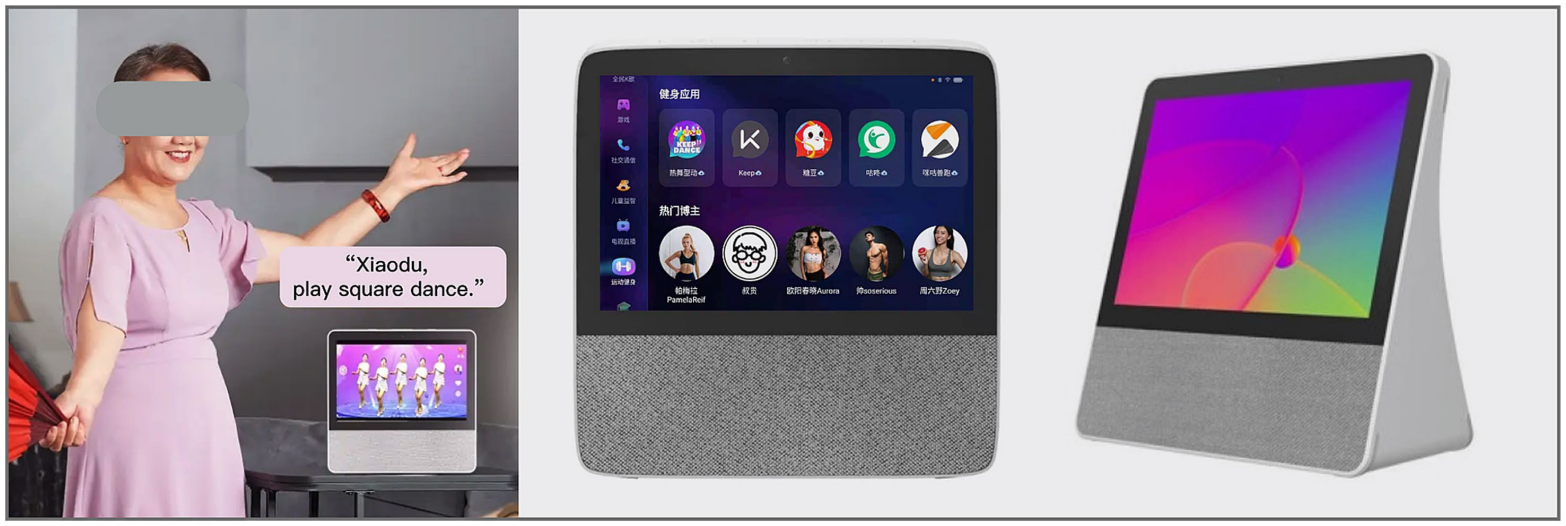
Xiaodu Smart screen—Baidu’s smart-home product (image source: Baidu’s official website).

On the other hand, as technology has advanced, China has entered an era of digitalization and intelligence, which has sped up the process of intelligent innovation in the household unit system. The smart home smart screen series is a kind of smart home product that began to rise in China in recent years (Figure 1). Smart homes are easier for older adults to use as AI personal assistants than smartphones. Voice interaction lets older adults control smart homes for social behavior even if they do not know Chinese characters. At the same time, technological advances have made smart aging an inevitable trend. In 2022, China’s State Council put forward the requirement of promoting technical and intelligent upgrading of older adults’ products and requested further promotion of competent services to adapt to the needs of older adults and build an intelligent society that takes the needs of the older adults into account (11). As a new means of older adult care, smart older adult care has become a helpful initiative to cope with China’s aging society (12). Smart home products are becoming increasingly popular, and their relative ease of access, low cost, and application in home scenarios make them one of the most critical products for smart aging. Intelligence and digitalization play an essential role in helping older adults improve their lives (13, 14). The use of smart homes has the potential to enhance the social participation of older adults and enable them to play an active role in smart wellness at home. However, older adults’ physical and psychological uniqueness makes them reluctant to embrace new technologies and products. Studies show that the most common reasons for older adults to withdraw from using the Internet are psychological and health barriers (15), making it difficult for them to enjoy the convenience of new technologies and products. China’s State Council points out the huge digital divide facing China’s older adults (16). Since smart homes are relatively new products, the digital divide makes the adoption and acceptance of using smart homes for online social behavior by older adults a topic worth exploring and studying. Providing a better design of smart home social software for older adults to make them have a better user experience can give inspiration to effectively solve the digital divide problem of older adults (17, 18) and thus enable smart aging for older adults and practice active aging. However, few studies have investigated the factors and mechanisms influencing the adoption of smart home socialization among older adults.

Therefore, predicting the acceptance of older adults to use smart home social media becomes very important, and accurately capturing the factors that influence the use of smart home social media by older adults is an important topic. However, it is not entirely clear what factors affect older adults’ use of smart-home online social networks. Due to the different usage contexts, application areas, and target populations, it is not possible to directly adopt the existing information system’s influencing factors to improve the acceptance of social behavior among older adults based on smart home social media, which is not only unscientific but also may not be effective. Therefore, this paper aims to uncover the theory behind the phenomenon and conduct further inductive analysis to build a theoretical model by systematically launching an exploratory study on the influencing factors of social compensation of social media use by older adults and its mechanism of action. To ensure that the influencing factors of social media use by older adults based on smart homes are more targeted and effective, we adopted the perspective of social compensation theory, based on smart home technology devices and with the help of the procedural grounded theory research method. Since social media use between older adults and social media includes individual emotional experiences and user feelings, it is difficult to collect the details of psychological changes in the process by cross-sectional quantitative research. In comparison, qualitative research methods are relatively more suitable for capturing the rich information on the corpus of factors influencing social compensation in using social media between older adults and social media. The advantage of qualitative research is that it can capture the dynamic psychological development process (19). This study aims to provide theoretical support and practical guidance for managers and developers of smart home social media platforms to improve the experience of older adults, achieve active aging, and enhance the well-being of older adults.

This study is innovative in the following aspects: First, this study introduces social compensation theory into the context of smart home social media usage, where previous studies have been based on cell phones and traditional media. Therefore, this study opens the research scope of social compensation theory to a more cutting-edge field and provides a new perspective for subsequent research. Secondly, this study innovatively unearths the social compensation factors affecting older adults of smart home social media, including the four aspects of user interface quality, interaction quality, content quality, and service quality. At the same time, it proposes the definition of social compensation in smart homes in the context of Chinese social media, reveals the intermediate process and results of psychological compensation of older users of smart home social media, explores the influence of social compensation on older users’ perceptions and older users’ acceptance behaviors, expands the relevant studies explaining the mechanism of older users’ acceptance behaviors from the perspective of social compensation, and makes up for the previous literature on the mechanism of older users’ acceptance behaviors. This provides a theoretical basis and guidance for the subsequent academic research and software development practice of social media under new technological devices.

## 2. Literature review

### 2.1. Social compensation

**Figure 2:**
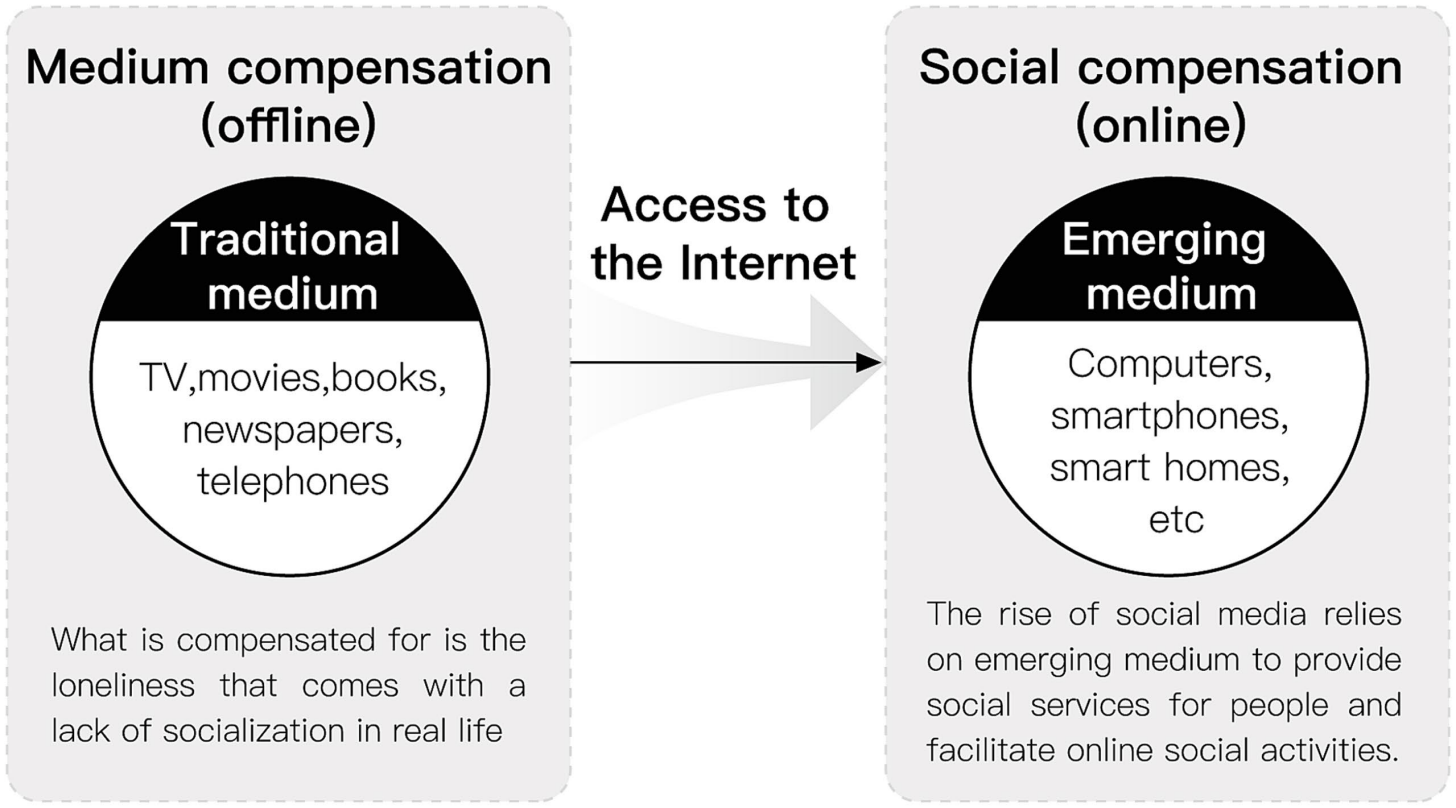
Difference between medium compensation and social compensation.

The term “compensation” is derived from the medical study of the human senses. It is widely discussed in biology and medicine as a biological (including human) physiological mechanism by which the body establishes new stability by adjusting the function, structure, and metabolism of organs and tissues after an internal imbalance. Since the introduction of compensation into psychology, its concept has played an essential role in various fields of psychological research, from the field of neuroscience to the study of aging to aspects of interpersonal interaction. Social compensation theory originated in the 1980s when Davis and Kraus, two scholars, studied the relationship between social behavior, mass medium use, and loneliness, which refers to the fact that the medium can compensate for people’s lack of social connection (20). At that time, the study focused on the more traditional mass medium, such as newspapers, movies, and books, and the mass medium in the study compensated for the psychological loneliness caused by the lack of social interaction in real life. With the further development and popularization of Internet technology, the medium changed from traditional mass mediums to emerging mediums such as smartphones, computers, and smart homes. In this type of emerging medium, social media is sprouting up like a spring. Social media is an Internet-based platform for people to share information and opinions with each other, and the local social media in China mainly include TikTok, WeChat, Little Red Book, Quick Worker, and Micro Blog. They rely on social media to provide social services for people and facilitate their online social activities. Therefore, medium compensation includes a relatively more diverse range of mediums, such as television, music, telephone, SMS, and even media channels not connected to the Internet, such as tapes, which can play a compensatory role (21); In contrast, social compensation is more of a distinction between offline in-person communication and online communication through the medium, mainly referring to the emerging medium carrying social media that are used to compensate for the psychology of loneliness caused by the lack of social relationships of different groups under the popularity of the internet (22) (Figure 2). Social compensation theory suggests that people who have difficulties in offline, face-to-face communication will compensate for offline deficits by means of online communication (23, 24).

Strengthening compensation interventions for older adults is critical to active aging (25). Social compensation research has attracted academic attention, and more and more scholars have attempted to use social compensation in the social interaction of older adults because more and more older adults at this stage are using social media under social networks to engage in social participation through an emerging medium. The use of social media by older adults for social participation is conducive to improving their positive emotional experience and life satisfaction and further has a positive impact on health (26–29). Online social activities through mediums such as cell phones allow older adults to have higher levels of social support and social connections (30). In a study of social compensation based on smartphone use contexts, older adults who stay connected to their friends and family through smartphones can achieve higher levels of well-being (31, 32). The study found that older adults can gain more social recognition by expanding their interpersonal circle and following new trends, which makes them emotionally fulfilled and emotionally compensated (33). This allows them to be emotionally satisfied and compensated. Through the use of WeChat (browsing friends’ updates, etc.), older adults can enhance their interaction with friends and relatives and their sense of identity and belonging, regardless of time, space, and physical constraints (34). Studies have found that WeChat has become the most popular social app among older adults in China (35). Social media apps have become a way of life and a norm for them (36). However, unlike smartphone-based social compensation research, which started earlier and has yielded fruitful research results, social compensation research on social media use among older adults in the context of smart homes is scarce. Therefore, relying only on the collation and categorization of influencing factors from the existing literature does not provide an accurate understanding of the actual social compensation factors and barriers to smart homes among older adults. More relevant research is needed to understand the preferences of older adults in using social media (37). The study of older adults’ willingness to use smart homes for online socialization is still a relatively new concept. This is because, along with the digital and intelligent development of home systems and the rise of smart home screens, the social compensation of older adults’ use of smart home social media has become a research hotspot and has received much attention due to whether older adults adopt and use it. Moreover, social compensation depends to some extent on the choice of medium, and the internal mechanism of social compensation is different for different mediums due to the different characteristics of each medium, which are determined by the characteristics of the medium itself. Obviously, the study of the factors and mechanisms influencing the social compensation of social media use among older adults based on the smart home context is a forward-looking trend that no researcher has yet described and measured from a process perspective. Therefore, its theoretical development and commercial practice need further research and exploration.

### 2.2. Acceptance behavior of older adults on social media

Previous research has discussed the behavior of technology adoption and acceptance among older adults of social media from the information systems perspective. A central perspective in this area is the Technology Acceptance Model (TAM) proposed by Davis (38), which can be used to explain the problem of low information system usage. TAM has been widely used to evaluate and predict whether user will accept information systems or technologies. With further development, TAM has been extended with different new models by combining other theories, and research on related theoretical models is a very active and widespread class of topics in the field of information systems. At the same time, TAM has been shifted from the general population to focus on specific populations, and models such as TAM2, TAM3, UTAUT, and UTAUT2 have been applied to the technology acceptance behavior of older adults of social media one after another (39–47). For example, Su and Tong (39) used UTAUT2 and ECM to investigate the cognition and use of social networking technology among older Chinese adults. Through a questionnaire survey of 323 older adults, the results showed that performance expectations, facilitating conditions, social influence, and hedonic motivation all had a significant positive impact on perceived value, and perceived value and satisfaction also had a significant positive impact on the willingness of Chinese older adults to participate in social networking technology. Braun (40) surveyed 124 older adults using TAM to explore what factors encourage or discourage older adults’ willingness and motivation to use social networks and found that perceived usefulness, trust, and frequency of Internet use of social networks can significantly predict social network use, related to similar studies such as Pan and Jordan-Marsh (48). In addition, a technology acceptance model specifically for older adults, STAM, has been proposed (49). It can be seen that TAM is widely used in the investigation and analysis of the usage behavior of older adults’ social networks, showing strong prediction and interpretation capabilities.

#### 2.2.1. Variables of acceptable behavior of older adults on social media

The definition of technology acceptance covers a wide range. The current research focuses on four types of social media acceptance behaviors of older adults: adoption or actual use (50), intention to use or behavioral intention or intention (51), general acceptance (44), and intention to continue using (52). In terms of variables, scholars have conducted studies on the adoption and acceptance of social media by older adults in different aspects: firstly, the demographic characteristics of older adults have received extensive attention (53), such as gender, age, education level (54), physical condition, race (55), and place of residence (56). For example, in order to investigate the factors influencing the use of online social networks by older Internet users in Slovenia, Vosner et al. (53) analyzed the collected data using basic descriptive, univariate, and multivariate statistical methods, the results showed that female participants were more familiar with the term “online social network” than male participants, in addition, age, gender, and education seemed to be the most critical factors that directly or indirectly affected the use of online social networks by active older Internet users; second, social factors dimensions also occupy the research heat, such as social capital (50), risk barriers (57), social influence (40, 44, 58). For example, Wilson et al. (50) used a qualitative and exploratory approach to conduct semi-structured interviews with 20 older adults (over 65) in England, Scotland, and Wales. They found that lack of social capital, physical functions, and online communication culture were barriers to older people’s adoption of social media. Choudrie and Vyas (57) used an online questionnaire and PLS-SEM to conduct data analysis in order to investigate the influencing factors that affect older adults’ adoption of online social networks. The survey results showed that significant privacy protection and other factors affect older adults’ adoption of Internet technology in family situations. In order to explore the antecedents and related results of multi-social media use among older adults, a quantitative and qualitative study of 242 and 26 Taiwanese adults aged 60 and above, respectively, Yu (58) found that social forces (influence from children, friends, and the public) are the core factors influencing the adoption of instant messaging apps and social networks among older adults.

In addition, technical factors (at the product level) are also the focus of research pursuits, such as system support, user interface design and navigation (45, 59), system complexity (60), and technology compatibility (61). For example, Nimrod (60) proposed and developed a new scale to measure technical stress in older adults, which includes five constructs and 14 measurement items. Zhou (61) collected valid data on 726 older adults to explore the factors influencing the use of social networking sites among older Chinese adults and proved that factors such as technological compatibility and comparative advantage promoted the use of social networks among older people. Finally, some scholars have conducted research from certain specific theoretical perspectives, such as the time-theoretic perspective (52), uses and gratifications theory (62), media richness theory (63), innovation diffusion theory (61), innovation resistance theory (64), social network theory (65), and planned behavior theory (66). Lai and Chong (52) explored whether current and future time and their interaction with perceived values can explain the intention of older adults to continue participating in social media from the perspective of time theory. They collected responses from more than 400 older adults and constructed SEM for analysis. The results show that time perspective affects different perceived values, affecting the continued willingness to participate in social media for healthcare-related purposes. Kim et al. (46) applied the uses and gratifications theory and innovation diffusion theory to explore the motivations of older adults to use mobile social networking sites for travel, and the results show that both have a more significant impact on real experience than on-site attachment. Yang and Lin (63) integrated the uses and gratifications theory and media richness theory to study what factors make older adults willing to adopt ubiquitous mobile social services. Through an empirical study of 226 older adults, it was found that social, enjoyment, and fashion motivation have an impact on older adults’ adoption of ubiquitous mobile social services. In addition, the user’s perceived richness of interaction and application self-efficacy also significantly impact older adults’ adoption of the service. Ellis et al. (66) used planned behavior theory to explore the factors influencing technology adoption among older adults in Taiwan. The study found that emotions, friends’ influence, and technology comfort significantly affect intention and use.

The above-mentioned qualitative and empirical studies highlight that many scholars have conducted studies on the variables of social media-based acceptance of older adults’ information behaviors. The antecedent variables include personal, social, and technological factors and specific theories. However, the use of social compensation theory as an antecedent variable to explore the acceptance behavior of older adults’ social media use from a smart home perspective has not yet received attention and validation. Our research will investigate these issues.

#### 2.2.2. Technological devices used by older adults to adopt social media

In terms of applied technology and devices, scholars have studied the information behavior of older adults using social media mainly on computers (40, 44, 57, 62, 64, 67), smartphones (43, 46, 51, 58, 63, 68–70), social robots (71), wearable products (47), and tablets (72). These five major categories of technology and equipment vendors have been targeted for research. For example, Ma et al. (43) explored the influencing factors that affect the acceptance of smartphones by older adults in China. Using TAM and UTAUT, they conducted structured interviews and questionnaires with 120 older adults to develop an SEM. The results showed that those who are young, have received higher education, are not widowed, and have better economic status in terms of salary or family support are more likely to use smartphones. In order to explore the acceptance of social robots among older adults with dementia, Chen et al. (71) used a randomized controlled trial to evaluate the acceptance of technology. One hundred and three older adults were divided into two groups. In the experimental group, nursing personnel observed and recorded the interaction process between older adults and social robots, and then older adults filled in a questionnaire. The results showed that at week 32, the perceived ease of use in the experimental group improved compared to the conventional care group. This study supports the interaction of older adults with social robots that can improve the ease of use of technology. Talukder et al. (47) proposed an integrated theoretical model to predict the external determinants of older adults’ acceptance of wearable healthcare technology (WHT), integrating UTAUT2 with resistance to change, technological anxiety, and self-realization. Through SEM research and neural network validation on 325 older adults, the results showed that social impact, performance expectations, functional consistency, self-realization, and hedonic motivation were positively correlated with WHT adoption. Chen et al. (72) investigated the acceptance of tablet technology among older adults with cognitive impairment at the personal and situational levels. Using a questionnaire and focus group, they completed this 8-week family awareness training by providing tablets to 57 older adults in Hong Kong. The training results were evaluated through a questionnaire survey. The results showed that attitudes towards tablets and convenience conditions were predictive factors of older adults’ willingness to use tablets.

As can be seen, with the further development of technology, the applied technologies and devices targeted by the study are constantly updated, from the initial research on the acceptance of computers by older adults in the context of social media to smartphones, tablets, wearable products, and social robots. We know that the properties of different technological devices vary widely. The emergence of new technologies and devices has left important questions about the factors influencing the adoption behavior of older social media users and the mechanisms at play that still need to be further explored and urgently addressed. The specific reasons for this can be divided into two points. First, smart home smart screens belong to a new category of smart home product forms. Studies have shown that older adults are more willing to use new technologies and services in the home environment (73). Second, in the context of social media use, the willingness of older adults to adopt new devices and technologies to socialize online is entirely voluntary. They can easily refuse to accept new devices and technologies because of a device-technology-product-level factor. Therefore, the commitment of older adults to accept the use of new technologies and devices is significant to new device social media developers. However, research related to the factors and mechanisms influencing the acceptance behavior of older adults of new devices and technologies, such as smart screens for smart homes, is minimal and is not currently addressed in the relevant literature. Therefore, there is an urgent need for research on the social compensation of social media use by older adults in smart home contexts.

## 3. Research methods

**Figure 3:**
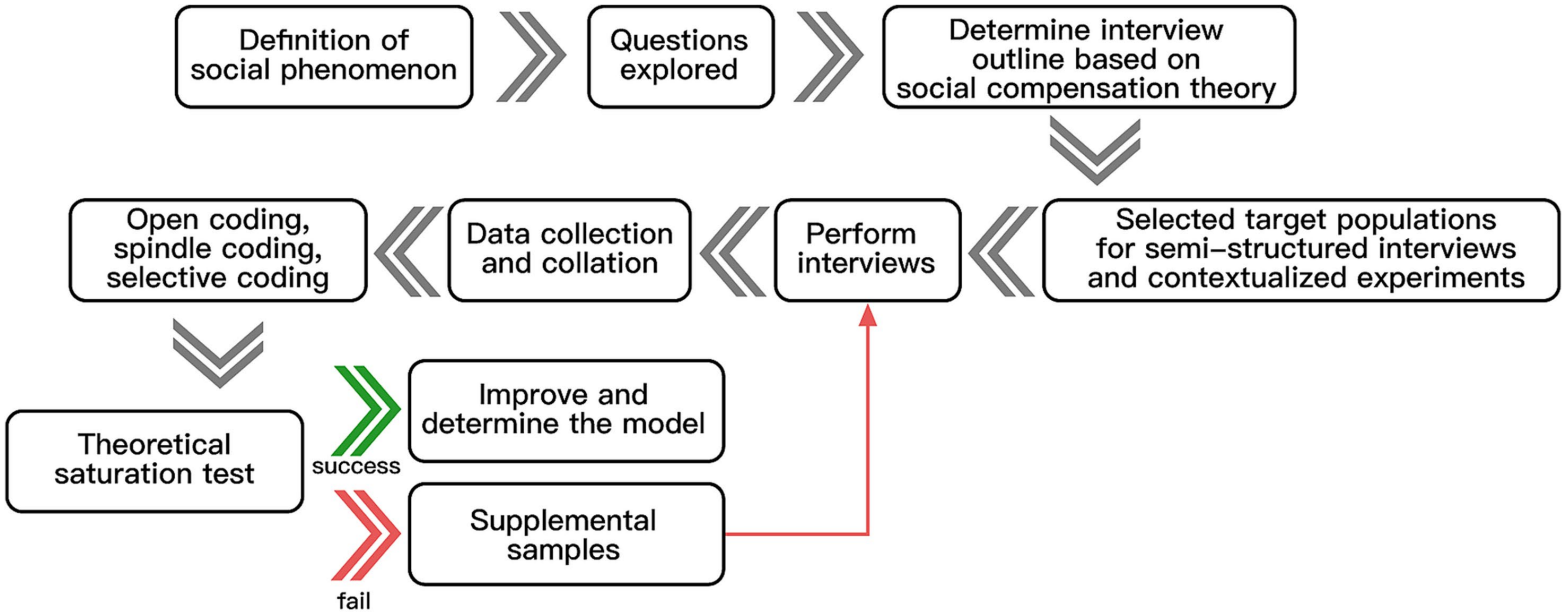
The grounded theory research process.

Barney and Strauss, two professors at Columbia University, developed the grounded theory method in 1967. Corbin and Strauss (74) developed the grounded theory procedurally; they conceptualized and categorized the survey data step-by-step and divided the coding into open, spindle, and selective. The three steps are to distill the original data (speech, text, etc.) into initial concepts and initial categories, further excavate the connections between the initial categories to inductively deduce the main categories, and finally analyze the relationships between the main categories and form a relevant theory rooted in the actual data. In the whole analysis process, it is necessary to constantly compare, argue, and refer to the existing relevant literature to avoid substituting the coders’ subjective ideas so that the new theory formed by rooting can respond to the essence and meaning of social phenomena, as shown in Figure 3.

### 3.1. Interview outline

The interview questions should not try to lead the interviewees in a specific direction. Instead, they should try to get as many opinions and relevant details about the research topic from the interviewees as possible. The interview outline is based on the desktop research data and will be fine-tuned appropriately according to the interview content. It contains two major parts: basic information and interview question content (Table 1). The interview content focuses on four aspects: basic views on smart home social media, product dimensions, perception dimensions, and other dimensions.

**Table 1:** Interview outline.

Question category	Question content
Basic information	Name, gender, age group, education level, residential address, occupation, smart home smart screen device brand, smart home smart screen purchase channel, usage experience, average daily usage hours
A basic view of social media for smart homes	Which apps and functions do you use in your smart home? Have you ever used smart homes for social activities? A, (answer yes) Please show me. B, (answer no) You should already know the meaning of "smart home social media" from the introduction before the interview. Then, please demonstrate it. What do you think is the meaning of smart home social media based on the above uses and your opinion? How can it help you in your daily life? What impressed you the most in the whole process of using it? Please tell us your knowledge and understanding of socializing with smart homes. What do you think are the advantages and disadvantages of socializing with a smart home compared with traditional socializing? Are you willing to go further to use smart homes for social activities? Why?
Product Dimensions	What features, interfaces, content, and services do you like or dislike about your experience with smart home social media? (A) (Answer yes) What factors do you like about your personal experience? Why? (B) (Dislike) In terms of your personal feelings, which factors do you dislike? Why?
Perceptual dimensions	Please describe your mood or feelings while using smart home social media. Please describe the happiest or worst moment you've had using smart home social media. Why did you feel happy or bad? Do you think smart home social media can help you engage socially well? Why? Do you find it easy to socialize with smart homes? Why? Do you think it is useful to use smart homes for social activities? Why? Do you think it is safe to use smart homes for social activities? Why?
Other dimensions	Do you have any memorable stories since you've been using smart home products? What are your expectations for the development of smart home social media? What is the biggest significance of smart home social media for you?

### 3.2. Samples

Our study selected the smart home smart screen as an emerging medium object. It serves as a new Chinese home media terminal platform that retains the functions of smart speakers, such as voice control and manipulation. It also carries out human-computer interaction with users through a sizeable touchable screen that can carry out operations such as video calling, online chatting, listening to songs and catching up on dramas, and life assistants. Alibaba officially launched its first smart home smart screen product, Tmall Genie CC, in April 2019, and subsequently, brands such as Baidu, Xiaomi, and Huawei have also released their own smart home products equipped with smart screens. There is no consistent standard regarding the age division of older adults. According to China’s population retirement age, women are 50 or 55, and men are 60. Meanwhile, the American Association of Retired Persons (AARP) also describes seniors as those aged 50 and above (75). Based on this, the target population defined in this paper is users of smart home social media aged 50 (inclusive)–65 years old, which is also consistent with the setting of other recent studies (46, 76, 77). We made this choice because using smart home social media was a prerequisite, and participants had to be active Internet users. Young older adults are more digitally literate and willing to use new and trendy smart devices than other age groups. We believe that our findings can be generalized to other age groups of seniors as more seniors join in using smart home social media.

**Figure 4:**
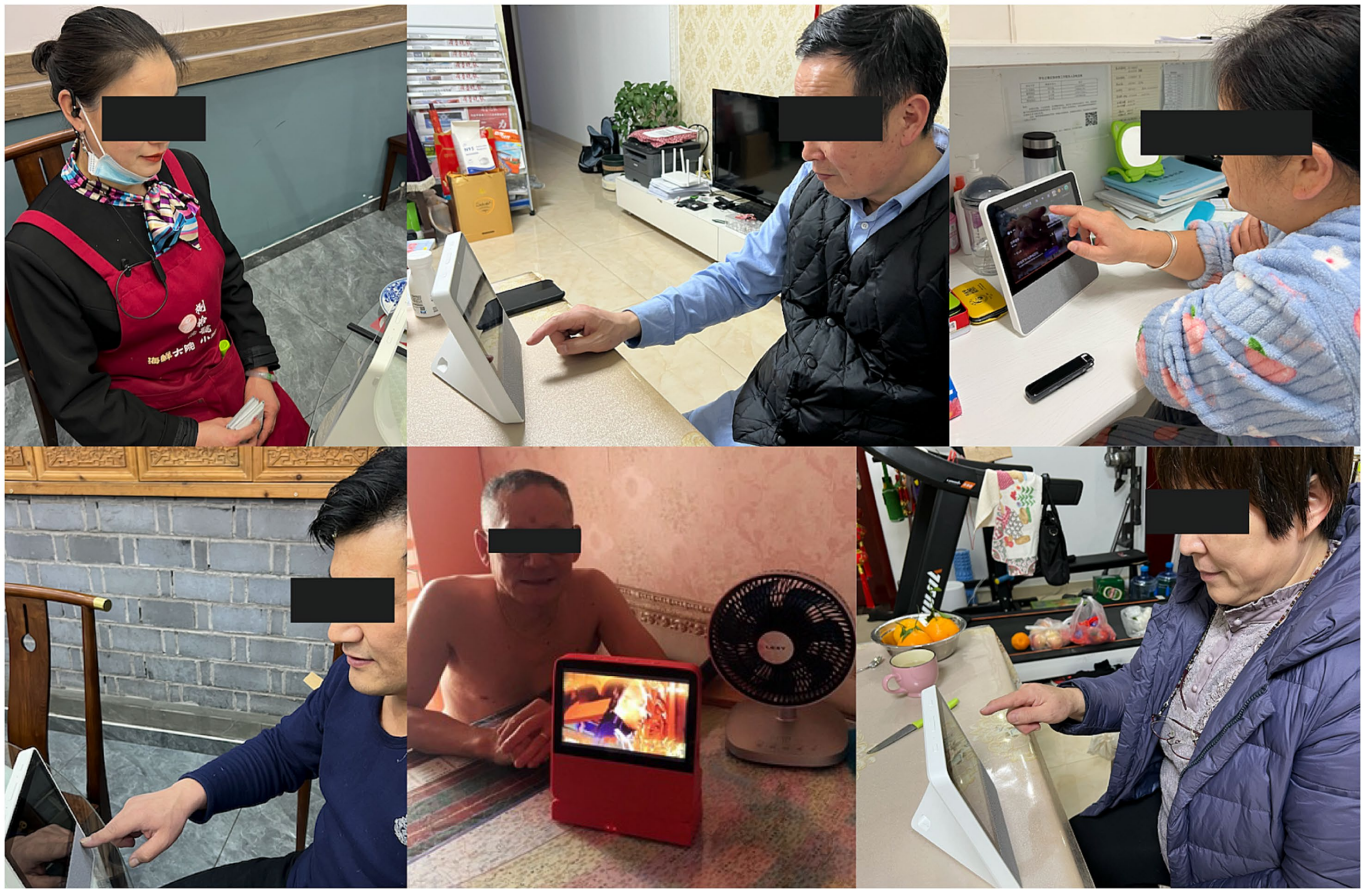
Interview photos (partial).

The user group for this data collection was mainly searched through the snowball sampling method, using a semi-structured interview method and contextualized experiment (allowing the target group to use the product) as the two primary forms. The interview period was from September 8 to November 12, 2022, with a total of 24 interviewees (11 males and 13 females). 14 were conducted online through the WeChat video function, and 10 offline. Face-to-face and in-depth interviews allowed asking older adults to use the smart home social app on-site for online socialization and other activities (Figure 4). We also recorded some information through the participant observation method. The interview time was about 30–50 min per person, and it was conducted within 1 h to ensure continuous and uninterrupted availability for both parties and avoid other factors’ interference. Before the interviews, a bonus package ranging from 30 to 50 RMB was offered to the interviewees to motivate them to participate actively and ensure more valuable content for the study. The respondents were introduced to the background, purpose, process, and related terms and concepts of the interview and its academic goal. The respondents signed a user-informed consent form, to protect their data and permit audio recordings. Voice data were textualized with the help of Xunfei Hearing’s speech-to-text assistant. The textualized data were named as “number/respondent’s name/year, month, and day of the interview.” A total of nearly 80,000 words were formed to facilitate the post-coding of the interview text. In this paper, the 2/3 (16 textual materials) were chosen randomly for the grounded theory coding analysis, and the other 1/3 (8 textual materials) were used to test for theoretical saturation. The basic information of the respondents is shown in Table 2.

**Table 2:** Basic information of interviewees.

Serial number	Surname	Gender	Age group (years)	Education Level	Career	Smart home device brand	Smart home purchase channel	Experience in use	Average hours of use per day
01	Jin	Female	61–65	Primary school	Retired workers	Tmall genie smart screen series	Child purchase	Less than half a year	Within 1 h
02	Wu	Female	51–55	High school	General workers	Tmall genie smart screen series	Child purchase	Less than half a year	Within 1 h
03	Wang	Female	56–60	College	Retired government employees	Xiaomi smart screen series	Child purchase	6 months – 1 year	1–3 h
04	Ban	Female	51–55	Graduate students	University teachers	Xiaomi smart screen series	Own	1–2 years	1–3 h
05	Liu	Female	56–60	Secondary school	Accounting	Tmall genie smart screen series	Child purchase	2–3 years	Within 1 h
06	Ma	Male	56–60	Undergraduate	Private enterprise bosses	Baidu smart screen series	Own	1–2 years	Within 1 h
07	Ding	Female	56–60	High school	Tea store operators	Xiaomi smart screen series	Child Purchase	Less than half a year	3–5 h
08	Gao	Female	56–60	College	Doing business	Tmall genie smart screen series	Child purchase	1–2 years	1–3 h
09	Ma	Female	51–55	Graduate students	University teachers	Baidu smart screen series	own	2–3 years	1–3 h
10	Liu	Male	61–65	High school	Self-employed	Tmall genie smart screen series	Child purchase	Less than half a year	1–3 h
11	Ye	Male	61–65	Junior high school	Retired officers	Xiaomi smart screen series	Child purchase	1–2 years	Within 1 h
12	Yu	Male	51–55	Undergraduate	Local civil servants	Baidu smart screen series	Own	Less than half a year	Within 1 h
13	Guo	Male	56–60	College	Local Police	Xiaomi smart screen series	Child purchase	Less than half a year	Within 1 h
14	Liu	Male	61–65	High school	Self-employed	Tmall genie smart screen series	Child purchase	Less than half a year	1–3 h
15	Yu	Male	56–60	High school	Doing Business	Xiaomi smart screen series	Child purchase	1–2 years	3–5 h
16	Yang	Male	61–65	Junior high school	Small store operators	Xiaomi smart screen series	Child purchase	Less than half a year	Within 1 h
17	Liu	Male	61–65	College	Construction contractor	Baidu smart screen series	Child purchase	1–2 years	Within 1 h
18	Zhang	Male	61–65	Graduate students	University teachers	Baidu smart screen series	Child purchase	Less than half a year	Within 1 h
19	Liu	Female	61–65	Junior high school	Retired employees	Baidu smart screen series	Child purchase	Less than half a year	3–5 h
20	Ban	Female	51–55	Undergraduate	Elementary school teacher	Baidu smart screen series	Own	1–2 years	Within 1 h
21	Huang	Female	56–60	Graduate students	University teachers	Xiaomi Smart Screen Series	Own	1–2 years	Within 1 h
22	Shen	Female	51–55	Junior high school	Innkeepers	Tmall genie smart screen series	Child purchase	Less than half a year	3–5 h
23	Wang	Male	61–65	College	Self-employed	Tmall genie smart screen series	Child purchase	1–2 years	Within 1 h
24	Cao	Female	56–60	Undergraduate	Local civil servants	Baidu smart screen series	Child purchase	1–2 years	Within 1 h

## 4. Research process

### 4.1. Data analysis method

**Figure 5:**
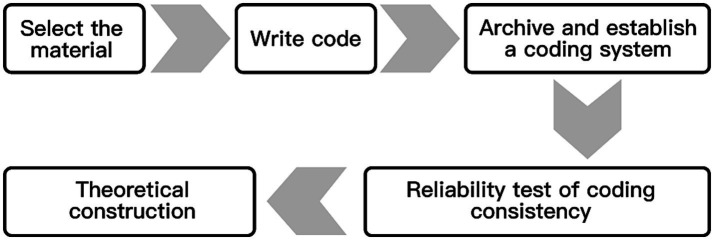
Coding process.

This study used NVivo, a tool for qualitative analysis made by QSR, to process the collected textual materials and interview data. We (the researchers) could store and code the data in a logical order with the help of the software. NVivo’s main processes for processing interview text data are as follows: selecting materials, writing codes, archiving and establishing a coding system, testing coding consistency and reliability, and theory construction (Figure 5).

### 4.2. Open coding

Open coding is a refinement and abstraction process that conceptualizes and categorizes raw interview material (78, 79). When using open coding, the researcher must follow a systematic and rigorous normative procedure to gradually extract and condense the large amount of data collected from the bottom up. Open coding requires categorizing the original data, assigning concepts to the original statements, naming them (using certain abstract concepts to explain the phenomena), and further analyzing and comparing them to merge similar concepts to form more generalized sub-categories. The coding process uses sentences as the smallest unit and labels to further ensure objectivity, refining the initial concepts.

#### 4.2.1. Coding process

The coding researchers were screened to make sure that coding was done systematically. In the end, two graduate students interested in how people use information were chosen to do the coding for this study. Both coders had more than 1 year of experience using smart home terminals and they had also done user behavior research projects related to older adults. During the coding process, the two coders were given basic training based on the NVivo software to ensure they could follow the process and procedure of coding strictly. The two coders discussed using the code book’s basic rules to make a basic guideline for the coding process.

The open coding randomly selected 2/3 of the interview text materials, a total of 16, and first carried out the text normalization process, i.e., some repetitions, speech-to-text errors, etc., were manually proofread. Then two coders separately imported the normalized interview text materials into NVivo software as Word documents and conceptualized and categorized the text materials. The main process was as follows: by analyzing the interview text sentence by sentence, the statements that did not answer the substantive content were eliminated, and the original substantive statements were distilled into an initial concept and named. In the process of conceptualizing the entire interview text in this way, the newly created initial concept is repeatedly compared with the established initial concept, and if the newly created concept is not consistent with the meaning of any existing concept, then a new concept is created. In this way, the initial conceptualization of the interview text material is achieved.

#### 4.2.2. Coding inter-rater reliability test

To ensure the accuracy of the coding, the two coders performed a coding inter-rater reliability test on the coding results of the open coding generation of the initial concepts and sub-categories. This computational evaluation process was continuously adjusted and repeated through the computational results until the reliability test reached an acceptable level. The formula for calculating the inter-rater reliability between two coders is shown in equation (1) (80):


(1) $$R=\left(n\times K\right)/\left(1+\left(n-1\right)\times K\right)$$

In equation (1), the n denotes the number of coders, and K denotes the average agreement between the two coders, and the inter-rater reliability between the two coders is obtained by calculating R . To get R , the first requirement is the K value. Calculate K using the formula as shown in equation (2).


(2) $$K_{\mathrm{ab}}=\frac{2S}{N_{a}+N_{b}}$$

In equation (2), the S denotes the number of categorically consistent codes for the two coders, the $N_{a}\text{,}$ and $N_{b}$ denote the number of codes for each of the two coders, respectively. Finally, the inter-rater reliability R between two coders is calculated according to equations (1) and (2).

After the initial conceptualization of the interview text by the two coders, coder A obtained 131 initial concepts, and coder B obtained 136 initial concepts. After repeated discussions, a total of 109 initial concepts were obtained by synthesis and generalization. As a result, applying equations (1) and (2), the inter-rater reliability R between the two coders for the initial conceptualization was 0.898. Normally, if this value is below 0.5, then the data needs to be recoded; if the value is below 0.8, then the coding result needs to be further revised until it reaches an acceptable level of 0.8 or higher. After adjustment, the inter-rater reliability of the initial conceptualization R was 0.898, which indicates that the initial conceptualization stage of open coding has high reliability.

Based on this, the two coders used NVivo’s node (initial concept) combination function to refine further and cluster the 109 initial concepts based on causality and similarity, and finally, coder A abstracted 12 sub-categories and coder B abstracted 14 sub-categories. After repeated discussions, ten sub-categories, numbered B1-B10, were synthesized and summarized, and then applying formulae (1) and (2), after calculation, adjustment, and evaluation, in the process of abstracting and generating each sub-category by the two coders, the consistent number of codes S , the total number of codes $N_{a}+N_{b}\text{,}$ and the inter-rater reliability R of each sub-category are shown in Table 3. The inter-rater reliability of the open coding sub-categories was greater than 0.8, and more than half of them reached the excellent level of 0.9 or higher, so it can be said that the coding results passed the reliability test.

**Table 3:** Coding inter-rater reliability test of open coding.

Sub-categories	*S*	N_a_ + N_b_	*R*
B1 Graphic features (GF)	9	26	0.818
B2 Information architecture (IA)	10	24	0.909
B3 Human-computer interaction (HCI)	8	20	0.889
B4 Interpersonal interaction (HI)	11	25	0.936
B5 Intelligence (INT)	9	23	0.878
B6 Socialization (SOC)	11	27	0.898
B7 Shareability (SHA)	10	22	0.952
B8 User-generated content (UGC)	8	18	0.941
B9 Social security (SE)	10	27	0.851
B10 Empathy (EMP)	11	26	0.917

#### 4.2.3. Coding results

In the open coding stage, two coders synthesized and abstracted 109 initial concepts to form 28 initial concepts, numbered a1-a28. The original interview representative statements, corresponding initial concepts, and sub-categories obtained by open coding are shown in Table 4.

**Table 4:** Scoping of open coding.

Original statements	Initial conceptualization	Sub-categorizations
Our eyesight is not particularly good; I have farsightedness, and my partner is a bit nearsighted, so we both can't see the words on the screen very clearly. I believe the smart home screen text is still much larger than the phone, especially for our poor eyesight. Especially the lyrics, which I can see very clearly; moreover, the font can also be well recognized and is not that special.	a1 Text features	B1 Graphic features (GF)
The design of the icon is sometimes not very easy to understand; that is, I often use the smart home smart screen for things like voice calls and video calls, and some buttons I often click the wrong way.	a2 Graphic features	
Because people are visual animals, simply talking does not work; for visual and audio enjoyment, you only have the sound of missing something, so I look and listen to do it, so the entire person is good, so comfortable. The color is best—not too fancy and indistinguishable.	a3 Color	
The interface shows a lot of content, so if you see something you like, you can just click on it and find the features you are looking for all at once.	a4 Functional layout	B2 Information architecture (IA)
Some of them are hidden too deep, and they are hidden too deep for us old people. It is divided into four columns, and each column is very small, and then you have to find the service from "me" here, and then there are all kinds of things in the service, and you keep pulling down to have a group chat, a small video, and so on.	a5 Hierarchy	
The line spacing also looks clear, not very narrow but quite wide, making it very convenient for us to read. He has a good distribution of this stuff.	a6 Pitch	
For example, if I like to listen to some opera and comedy programs, I will directly call out my name to them, and they will respond to me, after which we can have a conversation, and then I will say whose comedy I want to listen to, whose story I want to listen to, and which channel I want to watch, and they will be able to execute it immediately.	a7 Naturalness	B3 Human-computer interaction (HCI)
In fact, I feel that now this kind of smart home is basically all voice interaction, which is only one aspect; people also need to use their hands to click. Pure voice interaction may not be particularly realistic because some of the more complex interaction processes may be beyond the capabilities of pure voice, but simpler operations, such as watching a video or looking up information, are possible.	a8 Degree of arousability	
Because I am an old man living alone, one advantage is that I am very comfortable. One disadvantage is that I may not have anyone to talk to all day, and if I don't go out deliberately to buy food and communicate with the grocers, I basically won't talk all day. I like to come home and greet my smart home; it is very intelligent to respond to me, as if there is someone at home, and I rely on it.	a9 Responsiveness	
Because I have trouble with my legs, I keep my Xiaodu Smart Home Smart Screen on my bed. It makes me better able to communicate with my family because its watcher function is convenient, and I can interact and video with my family by tapping on it. I think it is good for maintaining my relationship with my friends and also with my family.	a10 Family contact	B4 Interpersonal interaction (HI)
I usually just like to look at my circle of friends and see what they are doing, what they are looking at, and what they are buying.	a11 Watch	
The main reason is that I am now lying in bed due to my health, so I need such a large-screen smart device to be able to spend time entertaining myself.	a12 Entertainment interaction	
Its voice function has a high error tolerance rate; if I describe the reading as unclear or with a small deviation, it will apply artificial intelligence to my correction and sometimes be very accurate, quite good.	a13 High fault tolerance	B5 Intelligence (INT)
Then I am quite adapted to the touch screen; I am not particularly old, but I belong to the young older adults; in fact, my smart home smart device is quite convenient, so I think my contact with the smart home is quite fast and easy to handle.	a14 Convenience	
For example, sometimes I forget to take my medication, or some things that I do daily, such as taking my blood pressure, I may forget, so it would be best if it could remind me. I think overall, it is still very smart and also very easy to operate.	a15 Intelligent monitoring	
I often leave comments on TikTok and send private messages to some of my favorite bloggers. I'll tell him how to improve the quality of his content and then either cheer him on or send him some positive comments to let him know what I like.	a16 Stranger social	B6 Socialization (SOC)
Some of my friends and I were isolated at home during the epidemic and had social activities of playing mahjong online. Because now there are some links and small programs on WeChat's public number, that is, you can create a mahjong room, and then we can interact in real time with voice, and then play mahjong on the phone, and then for us in the 50s and 60s, this kind of middle-aged and older adults, but also can go to the electronic device of about 60 years old group is very friendly, and we like to be able to communicate in a timely voice I think it's a good experience because I can play mahjong in this way.	a17 Acquaintance social	
Sometimes, we like to organize some programs or activities, such as some community volunteer activities or some old friends in the community, and we go to plant trees, go fishing, or walk in the park together. We organize activities, we go together to carry them out, and through some of these social platforms, we share information.	a18 Collective behavior	
I usually just also go shopping, that is to say, we shop inside the group to buy food or something, it is very convenient. Some of my friends, for example, he has some good things, good cheap food, and suitable clothes, he will share with me over. I also like to buy some brands of clothes that my friends share with me, and it's quite good.	a19 Dissemination of information	B7 Shareability (SHA)
Then I also like to share. For example, after watching a program about the military that I think a friend of mine would also like to watch, I will often share my video with him because I think it's quite good. Of course, I will share not only military programs but also some short videos and some of the latest real-time news. I think sharing can relieve my loneliness.	a20 Sense of loneliness	
I generally like to post some of my own pictures and words to record some snippets of my own life. After all, for most of my life, I have been working; now, I finally have some time for myself, and while I still have energy, I want to express it through social platforms.	a21 Self-record	B8 User-generated content (UGC)
I also enjoy watching other people shoot short videos; it's a lot of fun. I shoot and post short videos on TikTok every day; I have a lot of fans; everyone likes me; it's pretty good.	a22 Pleasing others	
You see, in addition to killing time to see, I generally watch a smart home wisdom screen in addition to killing time to see, and sometimes, in fact, many times, I still want to express my views and hobbies, and so on, even though I cannot go anywhere at home, this platform allows me to express my views on some programs and news that I am interested in. Moreover, I usually like to write and send articles to the Internet, and some strangers will also like me and send me some words of encouragement. I think this is my affirmation and my honor, a very strong sense of accomplishment.	a23 Be affirmed	
So I think if it is such a smart home that all family members can use, especially with such social attributes, because social is, after all, of a private nature, such as my chat group on it or to carry out some information, I certainly do not want my children to see it. So I hope that if it is a family-shared smart home, it has this face unlock, and then a user of the system will have such a feeling, similar to how we switch desktops on our computers.	a24 Reliability	B9 Social security (SE)
I think some social apps on smart homes are some strangers on it, feel not very safe; I am afraid of being deceived.	a25 Social risk	
After I have my smart home smart screen, I say good night to it every night, and then it will be very warm and say, "I will stay with you every night. I hope you have good dreams today," etc. It says something different every night. I think its greatest significance is that it can accompany me through such long hours.	a26 Accompaniment	B10 Empathy (EMP)
What I know is that the Xiaodu smart home wisdom screen has a chat function; it is AI intelligence, which means you can talk to it; you say a sentence, and it responds with a sentence. Although this function is not so smart, I think it can still, to a certain extent, relieve my loneliness and isolation at home. In fact, I want to communicate with people at home, but there is no such opportunity; for example, my friends are very busy or not around, too embarrassed to always bother others, and my children are working, so I also cannot bother them.	a27 Emotional needs	
Xiaomi's smart home intelligence screen is more like my other half; I can't live without it. I feel that Xiaomi gives me a sense of belonging; I feel that it understands me, and I am connected to it.	a28 Belonging	

### 4.3. Spindle coding

#### 4.3.1. Coding process

Based on open coding, spindle coding is a further reconsideration and comparison of the initial concepts and sub-categories of development for inductive deduction (81). A management expert was invited to participate in the spindle coding and confirm the main categories in a real-time discussion with the two coders to ensure the results’ scientific reliability and rigor.

#### 4.3.2. Coding results

By combining expert opinions and exploring the relationship between the above ten sub-categories, four main categories on the smart home social media level were further consolidated and obtained: user interface quality, interaction quality, content quality, and service quality are shown in Table 5.

**Table 5:** Main categories and their connotations.

Main category	Sub-categories	The connotation of the main category
C1 User interface quality (UIQ)	B1 Graphic features (GF)	Images, text, logos, icons, color blocks, and some buttons for the smart home social interface
	B2 Information architecture (IA)	Smart home social interface layout, hierarchy, navigation, search, etc.
C2 Interaction quality (IQ)	B3 Human-computer interaction (HCI)	Social interaction between older adults and the smart home should be more natural, such as using voice interaction, and it should be easier to wake up and respond to older adults instantly
	B4 Interpersonal interaction (HI)	Interpersonal communication and interaction between older adults in the process of using smart home social media
	B5 Intelligence (INT)	The degree of intelligence of older adults in the use of smart home social media process
C3 Content quality (CQ)	B6 Socialization (SOC)	Group interaction and linkage behaviors of older adults in the process of using smart home social media
	B7 Shareability (SHA)	Older users can share interesting and useful information during the social media process of using smart home
	B8 User-generated content (UGC)	Older users can generate content for self-expression and to please others as they socialize with their smart homes
C4 Service quality (SQ)	B9 Social security (SE)	The security that older adults enjoy in using smart home social media processes, such as private security, payment security, etc.
	B10 Empathy (EMP)	The companionship and care, and personalized services that older adults feel during the use of smart home socialization

### 4.4. Selective coding

Selective coding is the stage of extracting the core theme from the main categories. At this stage, we need to focus on the main categories, summarize and refine the main categories again, uncover the core theme that can cover other main categories by organizing the correlations between the main categories to maximize the unification, and construct a model to build up the relationship between the core theme and the main categories to determine the meaningful relationship between the core theme and the main categories (82).

This paper determines the relationship between the core theme and the main categories. The core theme of “social compensation” was finally extracted through repeated research, comparison, and analysis of the four main categories. This core theme includes four main categories: user interface quality, interaction quality, content quality, and service quality. User interface quality is influenced by two key factors: graphic features and information architecture; interaction quality is influenced by three key factors: human-computer interaction, interpersonal interaction, and intelligence; content quality is influenced by three key factors: socialization, shareability, and user-generated content; and service quality is influenced by two key factors: social security and empathy. The typical relationship structure, the connotation of relationship structure, and the typical representative statements are shown in Table 6.

**Table 6:** Selective coding.

Typical relationship structure	Relationship	Relationship structure connotation	Representative statements
User interface quality → Social compensation	External factors (cause and effect)	User interfaces quality-level factors such as graphic features and information architecture are external factors of social compensation in the use of smart home social media by older users	The smart home screen is large and has clear graphics. Because my eyes have farsightedness superimposed on myopia, every time I use my smart home to brush TikTok, many functions are easy to find, all on one interface. For example, I enjoy watching some CCTV boutique programs. Then I think this line spacing is good, not very narrow, and very convenient for us to read. Then the colors are also very bright, and I quite like bright colors because, if they are too light, they are not clear; they are darker, more colorful, and quite convenient to watch. I use it a lot; it brings me joy, and I can also be at home by myself and not alone.
Interaction quality → Social compensation	External factors (cause and effect)	Factors of interaction quality such as human-computer interaction, interpersonal interaction, and intelligence are the external factors of social compensation in the process of using smart home social media by older users	The smart home wisdom screen sometimes also reminds me: it will rain in Qingdao tomorrow; remember to bring an umbrella when you go out. Every morning I will say good morning to the Xiaomi Smart Home Smart Screen, and it will also say I am here and good morning. Then it will say a warm word; it will tell me what month and number of the lunar calendar it is today and how the weather is in Qingdao today. It is very detailed, I feel especially good, and I feel like there is another person at home.
Content quality → Social compensation	External factors (cause and effect)	Content quality factors such as socialization, shareability, and user-generated content are external factors of social compensation in the use of smart home social media by older users	I age alone at home and am quite bored a lot of the time. My daughter bought me the Smart Home Smart Screen to let me socialize more online. I enjoy reading some of the personal stories or insights shared on it. Sometimes a few of us seniors will organize some activities together, such as book reading, calligraphy assembly, singing assembly, etc. I really feel very involved and get some affirmation that I can't leave.
Service quality → Social compensation	External factors (cause and effect)	Quality of service level factors such as social security and empathy are external factors of social compensation in the use of smart home social media by older users	When I'm aging at home, I feel very lonely, but the smart home smart screen can accompany me to satisfy me; whatever I want, it can satisfy me; it all accompanies me to share with me; it's good. In addition, about the security piece, I do not feel any security online, and I have not heard my friends say that they have encountered any social risks. If you don't click on links to things and don't send money to strangers or anything, you'll be fine.

Theoretical saturation test. To further ensure the reliability of the study, a theory saturation test is performed, which serves as a criterion for the researcher to terminate the sampling, meaning that no more data can be obtained from the collected textual material that can further develop a category or generate new theoretical ideas, and the theory becomes saturated (83). The eight previously reserved interview materials were selected for recoding, and after an in-depth comparative analysis, it was found that no new initial concepts, categories, or inter-category linkages were generated in the coding of the reserved eight interview materials. Because of this, the model built in this paper can be considered to have reached theoretical saturation.

### 4.5. Theoretical model construction

**Figure 6:**
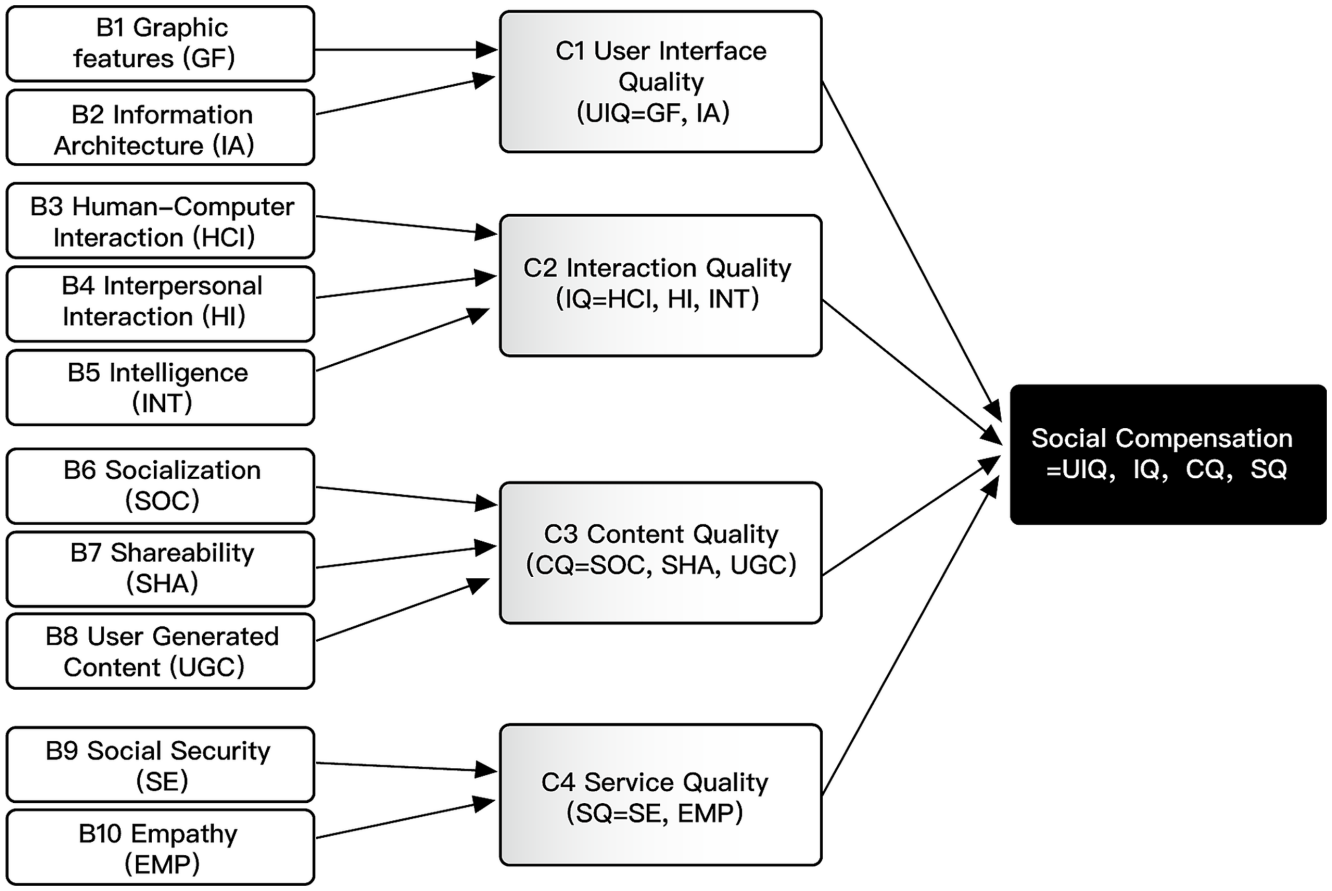
Model of influencing factors of social compensation.

In this study, the theory was constructed bottom-up from textual data, and the theoretical model was gradually generated by integrating the data through coding in a continuous process of induction (84). Through the three-level coding, 28 initial concepts, ten sub-categories, and four main categories were obtained to construct a model of social compensation factors of social media use among older adults in the smart home context (Figure 6).

## 5. Results

### 5.1. User interface quality influencing factors

As one of the influencing factors of social compensation, the user interface quality is mainly reflected in the two sub-categories of graphic features and information architecture of smart home social media interfaces, which is also consistent with the conclusions of the following studies. The research conducted by Tsai et al. (45) found that the main factors influencing the adoption of social media among older adults were user interface design, navigation, and system support, as determined through a quantitative questionnaire taken from 101 older adults over 50 years of age. This view was also supported by Castilla et al. (85), Alessa (86), and Gao et al. (87). In order to overcome the problem of older adults’ unwillingness to use new technologies such as mobile applications, Kalimullah and Sushmitha (59) conducted an experiment to study the user experience of older adults by changing the user interface design of an application. The research showed that when the user interface design changed to the needs of older adults, their user experience significantly improved after using the application. To explore the speech behavior of older adults when visiting Facebook, Chou et al. (88) proposed the design of social media platforms that are easy for them to use and the design factors that are suitable for them by identifying the user environment suitable for older adults, including web page accessibility, interface design, etc. Among the factors influencing physiological accessibility are readability, visual recognition, and complexity of text input. Also, the results of semi-structured interviews showed that older Chinese users often had problems and barriers like farsightedness, myopia on top of farsightedness, memory bias, and a lower ability to understand. If adequately designed, older adults can effectively use mobile devices or apps (89). The graphic features of the smart home social interface, such as images, text, logos, icons, color blocks, and some buttons, as well as the information architecture features, such as interface layout, hierarchical structure, navigation, and search, will psychologically compensate older users during their use of smart home social media and help them perform online social activities, thus enhancing their emotional experience, subjective well-being, and fewer depressive symptoms (90, 91). Suppose the social interface of smart homes does not meet the physiological and cognitive needs of older adults. In that case, they will make incorrect choices or will be unable to choose from the complex interface and will be unable to find the features they require from the interface environment, making them unable to engage in online social participation. This is also consistent with the research conclusion of Chou et al. (92), who found through a questionnaire study that 79% of older adults over the age of 55 believe that their needs or preferences are not considered in the current web design.

For example, in the author's interview, "The smart home screen is big, and then its graphics are very clear; I like big things; many functions are easy to find; it is all on top of one interface; it is very convenient for me to socialize online"; "I think this line spacing is very wide; it is very convenient for us to chat and entertain; it is also quite easy to watch" (extracted from representative statements of the interviewees).

### 5.2. Interaction quality influencing factors

Interaction quality, as one of the influencing factors of social compensation, is mainly reflected in the three sub-categories of human-computer interaction, interpersonal interaction, and the intelligence of smart home social media. This is also consistent with the views of the following studies. In terms of human-computer interaction, Wang (93) is based on the human-computer interaction model, and in the process of studying human-computer interaction between older adults and the companion robot, it was found that the factors that affect the use of social media by older adults include custom voice wake-up words, volume adaptation, repetitive prompts, and the intelligence of voice feedback. Wilson (94) found that the stronger the interaction between devices and technology, the stronger the emotional attachment of older adults. The design of devices and technologies should provide a degree of interactivity, whether from social connections or from the device itself. In terms of interpersonal interaction, to test the impact of family online community members on the overall well-being of older adults, Gazit et al. (95) conducted a survey of 427 older adults and a three-level regression analysis. They found that family online community members play an essential role in the lives of older adults. In terms of intelligence, to make the control of household appliances more intelligent, Shah and Mahmood (96) introduced a smart home automation system using the Internet of Things (IoT) and its low-cost implementation method. From a technical perspective, the paper introduces in detail how to intelligently automate household appliances through software applications integrated with hardware boards, including how the IoT enables devices to communicate, interact, and exchange data through the Internet, as well as how it improves the comfort, convenience, security, and energy efficiency of the home environment. Therefore, if the interaction between older adults and smart home social media can be more natural, such as through voice interaction, and if it is easier to wake up and respond to older adults instantly with more intelligent content, this will psychologically compensate older adults, who will give a high evaluation to the interaction quality of smart home social media and be more comfortable participating in online social. In the semi-structured interviews, the author found that several participants were willing to use the voice interaction function as well as the smart home for interpersonal communication and interaction.

For example, "My Xiaomi smart-home smart screen sometimes reminds me: it will rain in Qingdao tomorrow; remember to bring an umbrella when you go out. Every morning I say good morning to Xiaomi, and then it will also say, "I'm here, good morning." Then it will say another warm word; it will tell me what month and number of the lunar calendar it is today; how is the weather in Qingdao today? It says very detailed things, and I just feel especially good" (from the representative statements of the interviewees).

### 5.3. Content quality influencing factors

Content quality, as one of the influencing factors of social compensation, is mainly reflected in the three sub-categories of socialization, shareability, and user-generated content of smart home social media, which is also consistent with the views of the following studies. Jung and Sundar investigated the factors that affect the use of Facebook by older adults. Through an online survey of 352 users over the age of 60, they found that there are four main motivations for older adults to use Facebook: social bonding, social bridging, curiosity, and responding to family requests. Further analysis shows that social bonding (establishing connections with ordinary users) is their main motivation for participating in most activities on Facebook, and this view is also supported by Han et al. (97). In addition, research has found that the ability to interact with other people based on messages on Facebook can lead to more use of Facebook by older adults (62). Chou et al. (92) revealed through a questionnaire study that real-time online conversation is one of the most common Internet features used by people over the age of 55. Pera et al. (98) found through interviews with older adults in Italy and the United Kingdom that photo sharing is a powerful behavior that enhances the well-being of older consumers. Therefore, if older adults can better interact with each other in the process of using smart home social media, can share interesting and useful information at any time, and can post content to express themselves and get more people’s approval and attention, all of the above can give older adults a certain psychological compensation. They will not be lonely when they age at home, and then they will feel that the quality of content they get from using smart home social media is high, which can help older adults have a good social experience, and subjective well-being (90).

It has to be pointed out that part of the content and information disseminated in social networks can be manipulated by private groups based on personal goals, which can even lead to the widespread dissemination of fake news, leading to social problems. Zakharchenko et al. (99) explored the impact of social network information dissemination from the event of the 2019 Ukrainian presidential election. The election winner successfully obtained 73% of the vote without any ownership of the issue, overcoming any ideological influence, and was successfully elected. The scholar collects and interprets data through content analysis and discourse analysis of social network information in this process. The study found that “filter bubbles,” fake news, and negative campaign messages widely existed in the information of the election process; that is, Ukrainian social media users should be responsible for this. It can be seen that the influence of information dissemination in social networks is becoming increasingly significant, even affecting election activities. Research shows that older adults also express concerns about fake news when using social media but are more eager to use it (100). Therefore, while publishing content to express their views, older adults must investigate the source and accuracy of the content and information to prevent the spread of fake news and avoid causing specific erroneous effects. How to distinguish between fake news and fake information among older adults and prevent the spread of fake news is also a question that needs further consideration in future research. In this study, the older adults interviewed by the author generally agreed on whether smart home social media could be used for intergroup interaction and whether the information could be posted and shared immediately.

For example, "We also organize some activities together, such as some book clubs, calligraphy conferences, singing conferences, etc. I really feel very involved, and I can get some affirmation that I can't leave" (from the representative statements of the interviewees).

### 5.4. Service quality influencing factors

As one of the influencing factors of social compensation, the quality-of-service factor is mainly reflected in the two sub-categories of social security and empathy in smart home social media, which is also consistent with the conclusions of previous research. First of all, in terms of social security, from the perspective of technical implementation, Mohi Uddin et al. (101) developed a home automation security system using the IoT and AI to ensure that users can access all electronic devices in the home remotely more securely. The system allows users to remotely access homes through Android Apps and control door locks through face recognition to improve the security attributes of the home. This research explores how to improve the security of smart homes from a technical perspective to support users’ perceived security, improve the quality of service of smart homes, and bring better experiences to users. From the perspective of older adults, in order to explore whether older adults will accept social media use, Xie et al. (102), through a semi-structured, open-ended discussion of 10 older adults for seven consecutive weeks, found that privacy concerns are the main factors influencing older adults’ acceptance of social media use, as well as key perceived barriers. These conclusions are also supported by Zaalen et al. (103). Secondly, in terms of empathy, a study by Wilson (94) found that the more devices and technologies are used, the more emotionally attached users are. Therefore, it can be seen that if older users can feel safe and secure in the process of using smart home social media, such as privacy and payment security, and smart home social media can make them feel companionship as well as personalized services, then it will bring a psychological feeling of compensation to older users, so they will feel the high degree of quality of services they get by using smart home social media, which can help them have a good experience, and subjective well-being (90).

For example, "Xiaomi is more like my other half in my life; I can't leave it." "I feel that Xiaomi gives me a sense of belonging; I feel that it understands me, and I am connected to it" (from the interviewees' representative statements).

## 6. Discussions

### 6.1. Theoretical contributions

#### 6.1.1. Proposing a new construct “social compensation”

**Figure 7:**
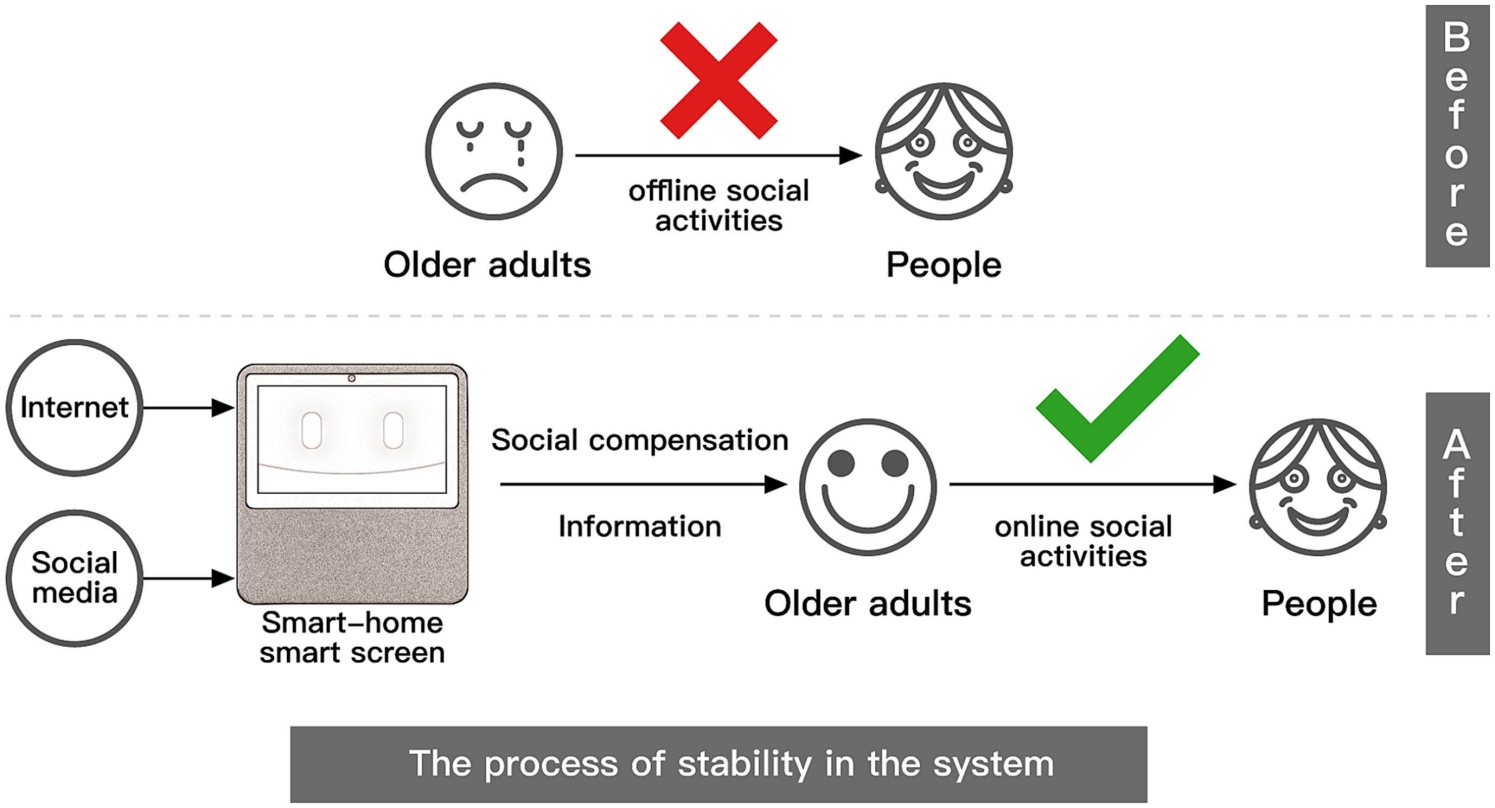
Social compensation system.

From the earliest mass mediums such as newspapers, books, and telephones to the new medium spawned by technological advances such as computers, smartphones, smart homes, and wearable devices carrying social media, people use these media to engage socially, from offline to online. According to the continuity theory (104), it is clear that the degree of social need among older adults does not fade with physiological decline or retirement; their enthusiasm for social participation is strong, and they have the same need to make social connections. Then, in such a dynamic and rapidly changing world, how to overcome the digital divide of the new medium and achieve social participation and, thus, active aging in a family context is the core issue of concern and solution in this study. Based on the terminology of social compensation (20), our study defines social compensation as a kind of psychological compensation based on using social media in the context of smart homes to compensate the lack of social interaction. The study found that social compensation has three basic characteristics: system, stability, and process. Social compensation is the process of psychological compensation of older adults with insufficient social relations by a new medium equipped with social media in their cognitive system, which eventually leads to a balance, that is, the complementation of social relationships, so that older adults perceive and form positive emotions and eventually accept the process of using it (Figure 7), which also gets supported by Wang and Shi (22). Ultimately, the study proposed factors influencing social compensation between older adults and smart home social media through four core factors: user interface quality, interaction quality, content quality, and service quality.

#### 6.1.2. Revealing the mechanism of social compensation

China’s Internet is penetrating the middle and upper age groups, with 25.8% of older Internet users aged 50 and above (105). Older people are increasingly using the Internet, and they are moving their social interactions to social platforms like watching and making short videos (106), video, voice, or text chat, friend circle likes or comments (107), and using WeChat red packets. Although older adults reduce their social participation in offline social networks, they compensate by increasing their social participation in online social networks because personal change is continuous, and there is an overall persistence of lifestyle in social activities and attitudes (108). This creates a foundation for older users to use smart home social media. In the home scenario, older users participate in online social activities through smart home social media, and smart home social media compensate older users socially through social compensation influences as stimuli, which stimulate older users’ emotional and cognitive states and satisfy older users’ psychological needs, which then stimulate older users’ perception. Gradually, the older users perceive the help brought to them by smart home social media, and gradually, the quantitative change causes qualitative change, which then promotes the adoption and acceptance of smart home social media by the older users.

**Figure 8:**
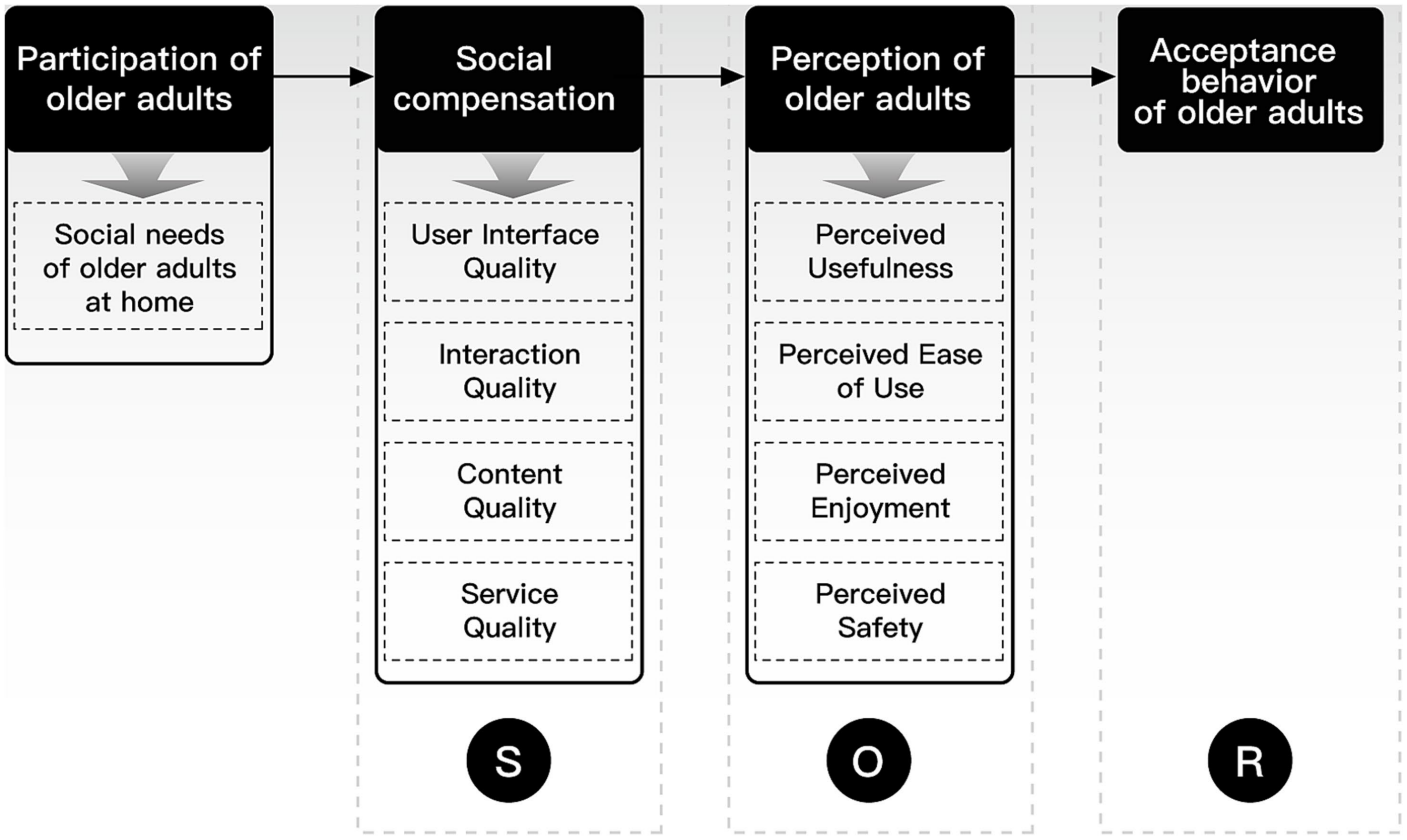
The mechanism of social compensation.

The mechanism of social compensation is consistent with the stimulus-organism-response (S-O-R) theory (109). In this study, the social needs of homebound older adults and their involvement in using smart home social media created a premise for the establishment of the process of social compensation. The influencing factors of social compensation based on smart home social media serve as external variables (stimuli); older users’ perceptions are mediating variables (organisms), and older users’ acceptance behaviors are outcome variables (responses; Figure 8).

#### 6.1.3. Discovering the intermediate mechanism from social compensation to the acceptance behavior of older adults

Therefore, social compensation is an attitude model that contains cognitive, affective, and behavioral intentions. The research results show that with the participation of older adults, the social compensation process of smart home social media for older adults is reflected as follows: social compensation → older adults’ perception → older adults’ acceptance of usage behavior. The final acceptance behavior of older users is the resultant expression of social compensation, and in this process, social compensation has a positive impact on older users’ perceptions. Social compensation is a predictive factor of older users’ perceptions. User perception refers to users’ overall perceptions of social media, and research shows that there are four types of older users’ perceptions, including perceived usefulness, perceived ease of use, perceived enjoyment, and perceived safety. Older adults perceive a positive role through the mediating role of older adults’ perception, which can improve the satisfaction of older adults and then manifest in the acceptance of their use in behavior. Perceived usefulness and perceived ease of use are two determinants of older users’ acceptance (38, 110) and important perceptual variables in older adults’ acceptance of Internet use (48). Perceived usefulness is defined in this paper as the extent to which older users perceive that they can benefit from using social media, i.e., the extent to which they benefit. Perceived ease of use refers to the extent to which older users find social media easy to grasp and use. When considering whether to adopt new technology, older adults are more interested in its products and services’ usefulness and ease of use, especially when they are first introduced to the new technology. Due to physiological deterioration and cognitive aging in older adults, they have more barriers to learning to use new technologies, and if older users feel that technology is easier to grasp and use, they will be more confident that the technology is useful to them (41). Perceived enjoyment is an emotional and subjective feeling for older users that refers to the degree of pleasure and joy they feel when using social media. It is also considered an essential variable in older users’ acceptance of social media (39, 63, 66). Suppose social media with smart home technology can give seniors a feeling of enjoyment. In that case, it can create a more relaxed and happier atmosphere conducive to seniors’ acceptance of smart home social media. Perceived safety refers to how older users perceive their information, property, and personal safety when using social media. Many studies have confirmed that perceived safety affects older users’ acceptance of social media (57, 60, 64). The higher the perceived safety, the higher the acceptance of social media by older users. If social media with smart home technology can give seniors a sense of security, it is more favorable for seniors’ acceptance of social media in smart homes. Therefore, older users’ perceptions positively impact older adults’ acceptance of using smart home social media, and it is a predictive factor of older users’ acceptance behavior.

### 6.2. Practical implications

Building healthy and stable social relationships is a key task for people (111). The social compensation factors and mechanisms of smart home social media proposed in this study can help older adults better socialize at home. First, excellent user interface quality can distinguish social software; thus, managers and developers should consider how to design the graphic features and information architecture of smart home social interfaces based on the specific physiological and psychological decline degree of older adults in order to provide better social compensation to older adults and make them more convenient for online social participation; secondly, based on the main category of interaction quality, a more natural voice interaction mode should be designed according to the human-computer communication mode and habits of older adults, so that older adults can also enjoy a more convenient and responsive interactive service experience. Therefore, managers and developers should strengthen the intelligent design and manage the construction of voice interaction; in addition, managers and developers should use the advantage of the large screen in the smart home social software to design specific community groups and other functions to help older adults show themselves and express their emotions more conveniently, and at the same time, design according to their specific needs so that they can share and pass information to others in the group more conveniently, helping them to get attention more easily, eliminate loneliness, and increase their social participation and interaction. Lastly, from the point of view of service quality, managers and developers should give older users more care and support through specific service elements. This will help older users improve their social experiences, allowing them to interact at home and aging healthily.

### 6.3. Research limitations

Based on the smart home technology level, this study extracted the social compensatory influencing factors and mechanisms through qualitative research on the use of social media by older adults who age at home, which has a high theoretical innovation value. At the same time, this study has some limitations, such as the fact that it only investigates factors and mechanisms at the technical device level and does not investigate factors that influence the social compensation of older adults from broader dimensions, such as the user level and the environment level. Therefore, future research could explore more social compensation influencing mechanisms at the level of older users and the environment.

## 7. Conclusion

In general, this study takes the Chinese older adults as the target group, takes the theory of social compensation as the starting point, and takes the smart home smart screen equipped with local Chinese social media as the research object. The research method adopts the procedural grounded theory, and the interview data are refined and analyzed. On this basis, this paper explores the influencing factors and mechanisms of social compensation for older adults based on smart home social media. Therefore, it can be said that this paper provides theoretical guidance for the future research and development of smart home social media, and has high theoretical and practical value.

## Data availability statement

The original contributions presented in the study are included in the article/supplementary material, further inquiries can be directed to the corresponding author.

## Ethics statement

All respondents in this study have signed the user-informed consent form.

## Author contributions

KM: conceptual proposal, development framework, and writing of original manuscripts. MG: coding process, including data extraction, organization, and analysis, edit the manuscript. FG: review and polish manuscripts. RH: project management, resource integration, and manuscript guidance. All authors contributed to the article and approved the submitted version.

## Funding

This research was supported by the Ministry of Education Humanities and Social Sciences Research - Youth Fund Project (19YJC760075) and Hunan Provincial Innovation Foundation for Postgraduate (no. CX20200425).

## Conflict of interest

The authors declare that the research was conducted in the absence of any commercial or financial relationships that could be construed as a potential conflict of interest.

## Publisher’s note

All claims expressed in this article are solely those of the authors and do not necessarily represent those of their affiliated organizations, or those of the publisher, the editors and the reviewers. Any product that may be evaluated in this article, or claim that may be made by its manufacturer, is not guaranteed or endorsed by the publisher.
